# Formation of strength platform in cast Al–Si–Mg–Cu alloys

**DOI:** 10.1038/s41598-019-46134-7

**Published:** 2019-07-03

**Authors:** Xixi Dong, Sajjad Amirkhanlou, Shouxun Ji

**Affiliations:** 1 Brunel Centre for Advanced Solidification Technology (BCAST), Institute of Materials and Manufacturing, Brunel University London, Uxbridge, UB8 3PH United Kingdom; 2 Department of Materials, University of Oxford, Oxford, OX1 3PH United Kingdom

**Keywords:** Metals and alloys, Mechanical engineering

## Abstract

Over the past several decades, it was generally believed that the strength of the industrially widely used cast Al–Si–Mg–Cu alloys enhanced monotonously with increasing Cu content. However, in this study using cast Al9Si0.5MgxCu (x = 0,0.2,0.4,0.6,0.85,1.0,1.25, in wt.%) alloys under T6 heat-treated condition, it was found that the hardness and yield strength of the heat-treated alloys showed a platform in the composition range from 0.4 wt.% to 0.85 wt.% Cu, while still increased with increasing Cu content before and after the platform. With increasing Cu content, the β-Mg_2_Si intermetallic phase decreased and disappeared at 0.85 wt.% Cu, while the Q–Al_5_Cu_2_Mg_8_Si_6_ and θ–Al_2_Cu intermetallic phases increased in the as-cast alloys. After heat treatment, the micron-scale intermetallic phases were dissolved into the Al matrix and precipitated as the nanoscale β″, Q′ and θ′ strengthening phases. With increasing Cu content, the β″ precipitate decreased and vanished at 0.85 wt.% Cu, while the Q′ and θ′ precipitates increased in the heat-treated alloys. The trade-off of the phases induces the platform in the strength of the heat-treated alloys, and further increase of the Cu content in this undetected trapped platform range is not favorited industrially as it only decreases ductility.

## Introduction

Cast Al–Si–Mg alloys have been widely used in industry, in particular in automotive, rail transit and aerospace industries for the manufacturing of light-weight structures due to the excellent combination of good castability, strength, ductility, and corrosion resistance^1^. The cast Al–Si–Mg alloys can be significantly strengthened after T6 heat treatment including solid solution and artificial ageing, due to the precipitation of the nanoscale β′′–Mg_2_Si strengthening phase, and the alloys are mainly used after T6 heat treatment^2–4^. In order to strengthen the cast Al–Si–Mg alloys further, Cu is usually added into the cast Al–Si–Mg–Cu alloys^5–7^.

Studies have extensively investigated the mechanisms of strengthening in wrought Al–Si–Mg–Cu alloys after T6 heat treatment^8–15^. However, the research results in wrought Al–Si–Mg–Cu alloys could not be simply transplanted to the cast Al–Si–Mg–Cu alloys, due to the significantly increased solute contents in cast alloys. The effects of Cu content on the mechanical properties and the strengthening of the T6 heat-treated cast Al–Si–Mg–Cu alloys are still unclear, which results in the blind addition of Cu in the alloys.

Over the past several decades, based on the insufficient investigating Cu content with a coarse interval of at least 0.5 wt.%, it was generally believed that the strength of the T6 heat-treated cast Al–Si–Mg–Cu alloys increased monotonously with the increase of Cu content^16–19^. Different phases including the Cu-free β′′ precipitate and the Cu-containing Q′–Al_5_Cu_2_Mg_8_Si_6_ and θ′–Al_2_Cu precipitates were reported for the strengthening of T6 heat-treated cast Al–Si–Mg–Cu alloys under sporadic Cu contents^17–22^, while the effect of Cu in the refined composition range on the strengthening of the alloys is still scientifically blind.

Based on the above mentioned non-rigorous believing, it is the present practice pursuing the strengthening of the cast Al–Si–Mg–Cu alloys by adding more Cu with the sacrifice of part of the ductility, as the ductility of the alloys was accepted decreasing with increasing Cu content due to the increase of defect level^6,16^. However, the trade-off of the Cu-free and Cu-containing strengthening phases with increasing Cu content and their possible balancing effect to strength in specific composition range were hardly noticed, and the blind addition of Cu might fall into the trap of the deterioration of ductility without any enhancement in strength.

In this study, using the T6 heat-treated cast Al9Si0.5MgCu with a refined Cu content interval of ~0.2 wt.% in a wide composition range up to 1.25 wt.% Cu, the strength platform in cast Al–Si–Mg–Cu alloys was revealed and discussed, which supplements the scientific understanding of the positive correlation of Cu content and strength and sheds a light on the scientific choice of Cu content in the industrially widely used cast Al–Si–Mg–Cu alloys.

## Results

### Mechanical analysis

**Figure 1:**
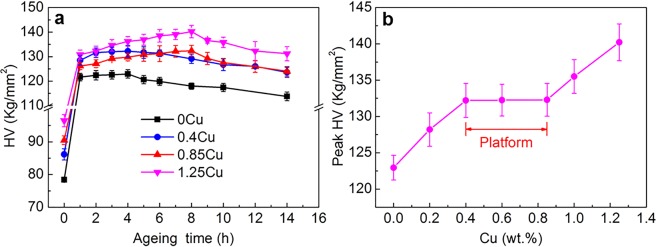
The hardness of cast Al9Si0.5MgCu alloys with different Cu contents: (**a**) the hardness of Al9Si0.5MgCu alloys under different ageing time after solution treatment and (**b**) the peak hardness of Al9Si0.5MgCu alloys after T6 heat treatment.

Figure 1a shows the evolution of the Vickers hardness (HV) of the cast Al9Si0.5MgCu alloys with ageing time after solution treatment. With increasing ageing time, the hardness of the alloys first increased due to the precipitation of the small nanoscale metastable precipitates that had strong strengthening capability, and then the hardness decreased due to the growing of the precipitates into stable phases that had relatively weak strengthening effect. The hardness of the 0 wt.% and 0.4 wt.% Cu alloys reached the peak at an ageing time of 4 hour, while the hardness of the 0.85 wt.% and 1.25 wt.% Cu alloys reached the peak at an ageing time of 8 hour. The solution and peak ageing treatment was used as the process to evaluate the strength of the alloys, as it provided the maximum capability of the strength. In the following text, T6 heat treatment represents solution and peak ageing treatment. Figure 1b presents the evolution of the peak hardness of the Al9Si0.5MgCu alloys with Cu contents after T6 heat treatment. The peak hardness of the alloys increased with increasing Cu content. However, a platform was clearly observed between 0.4 wt.% and 0.85 wt.% Cu, which indicated that the increase of Cu content in the range of 0.4 wt.% and 0.85 wt.% Cu did not increase the peak hardness of the T6 heat-treated Al9Si0.5MgCu alloys.

**Figure 2:**
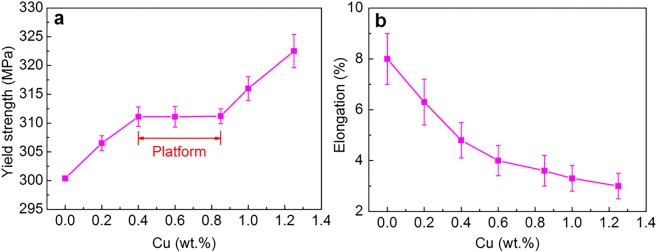
The tensile properties of cast Al9Si0.5MgCu alloys with different Cu contents after T6 heat treatment: (**a**) yield strength and (**b**) tensile elongation.

Figure 2 presents the tensile properties of the cast Al9Si0.5MgCu alloys with different Cu contents after T6 heat treatment. From Fig. 2a, the yield strength of the alloys also kept as platform between 0.4 wt.% and 0.85 wt.% Cu, and increased with increasing Cu content before and after the platform. The strength of the T6 heat-treated cast Al–Si–Mg–Cu alloys doesn′t enhance with increasing Cu content between the specific composition range of 0.4 wt.% Cu and 0.85 wt.% Cu. From Fig. 2b, the ductility of the alloys decreased with increasing Cu content. The further increase of Cu content in the platform range is not favorited as it only decreases ductility.

### Microstructural analysis

#### As-cast microstructure

**Figure 3:**
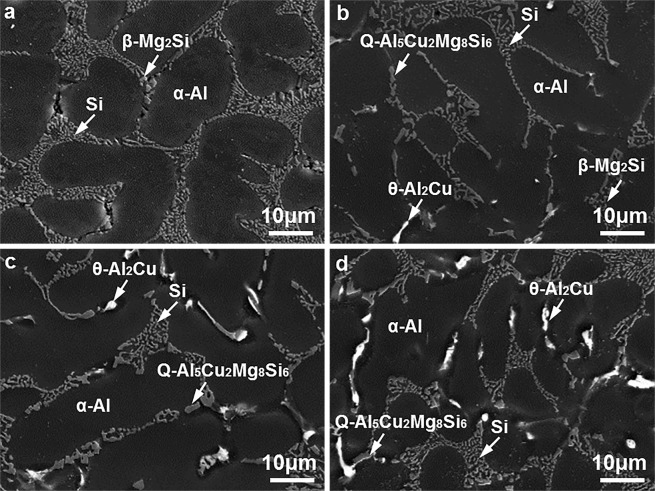
SEM micrographs showing the microstructure of the as-cast Al9Si0.5MgCu alloys with different Cu contents, (**a**) 0 wt.% Cu, (**b**) 0.4 wt.% Cu, (**c**) 0.85 wt.% Cu and (**d**) 1.25 wt.% Cu.

**Figure 4:**
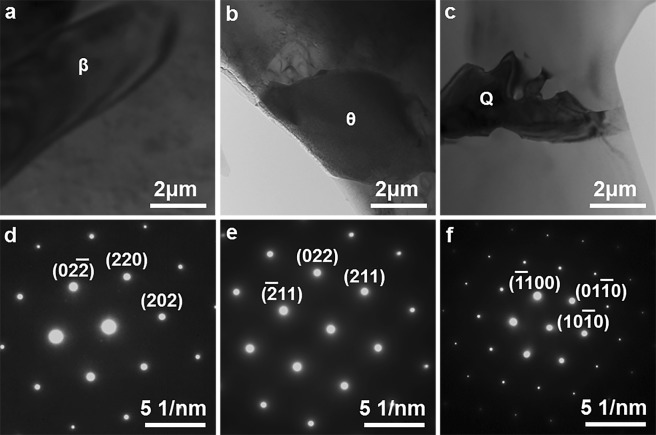
(**a**–**c**) Bright-field TEM micrographs and (**d**–**f**) selected area electron diffraction patterns confirming the intermetallic phases in the as-cast Al9Si0.5MgCu alloys, (**a**,**d**) β–Mg_2_Si phase, (**b**,**e**) θ–Al_2_Cu phase and (**c**,**f**) Q–Al_5_Cu_2_Mg_8_Si_6_ phase.

For brevity, the microstructure of the Al9Si0.5MgCu alloys at the key points of 0 wt.% Cu, 0.4 wt.% Cu, 0.85 wt.% Cu and 1.25 wt.% Cu is presented in the following text. Figure 3 shows the microstructure of the as-cast Al9Si0.5MgCu alloys with different Cu contents under SEM. The as-cast microstructures of the alloys consisted of the primary α–Al matrix phase, and the eutectic Si phase and the intermetallic phases in the grain boundary. For the alloy without Cu, β–Mg_2_Si was the intermetallic phase, as shown in Fig. 3a. After adding Cu, the Cu-containing Q–Al_5_Cu_2_Mg_8_Si_6_ and θ–Al_2_Cu intermetallic phases were observed in the as–cast microstructures, as shown in Fig. 3b–d. With the increase of the Cu content, the volume fractions of the Q and θ intermetallic phases increased, while the volume fraction of the β intermetallic phase decreased, and the β intermetallic phase was hardly observed when the Cu content was increased to 0.85 wt.%. The β, Q and θ intermetallic phases observed under SEM were determined precisely by TEM, as shown in Fig. 4.

The morphology, contrast and rough composition of different phases can be distinguished under SEM. However, SEM is unable to provide a precise confirmation of phases as it can′t reveal the inherent lattice structure of phases, and TEM is needed to determine the β, Q and θ intermetallic phases observed under SEM. Figure 4a–c present the bright-field TEM images of the β, Q and θ intermetallic phases in the as-cast Al9Si0.5MgCu alloys orderly. Figure 4d–f show the corresponding selected area electron diffraction patterns of the β, Q and θ intermetallic phases in Fig. 4a–c, respectively. The lattice structure of the β intermetallic phase is face-centered cubic (FCC) with the unit cell parameter of *a* = 0.6351 nm, and the consequent interplanar spacing of the (202) planes of the β intermetallic phase is 0.2245 nm. The selected area electron diffraction pattern shown in Fig. 4d was observed from the zone axis of [−111] and indicated a 0.2235 nm interplanar spacing of the (202) planes, which agreed well with the interplanar spacing of the (202) planes of the β intermetallic phase, and it confirmed that the intermetallic phase shown in Fig. 4a was the β phase. The lattice structure of the θ intermetallic phase is body-centered tetragonal (BCT) with the unit cell parameters of *a* = 0.6066 nm and *c* = 0.4874 nm, and the consequent interplanar spacing of the (211) and (022) planes of the θ intermetallic phase are 0.2370 nm and 0.2144 nm, separately. The standard angle of the (211) and (022) planes of the θ intermetallic phase is 54.7°. The selected area electron diffraction pattern shown in Fig. 4e was observed from the zone axis of [0–11], and it indicated a 0.2357 nm interplanar spacing of the (211) planes and a 0.2125 nm interplanar spacing of the (022) planes, which agreed well with the interplanar spacing of the (211) and (022) planes of the θ intermetallic phase. In addition, the measured angle of 54.5° between the (211) and (022) planes in Fig. 4e also agreed well with the standard angle of the (211) and (022) planes of the θ intermetallic phase. Thus the selected area electron diffraction pattern shown in Fig. 4e confirmed that the intermetallic phase shown in Fig. 4b was the θ phase. The lattice structure of the Q intermetallic phase is hexagonal close pack (HCP) with the unit cell parameters of *a* = 0.10393 nm and *c* = 0.4017 nm, and the consequent interplanar spacing of the (10-10) planes of the Q intermetallic phase is 0.3402 nm. The selected area electron diffraction pattern shown in Fig. 4f was observed from the zone axis of [0001] and indicated a 0.3389 nm interplanar spacing of the (10-10) planes, which agreed well with the interplanar spacing of the (10-10) planes of the Q intermetallic phase, and it confirmed that the intermetallic phase shown in Fig. 4c was the Q phase.

**Figure 5:**
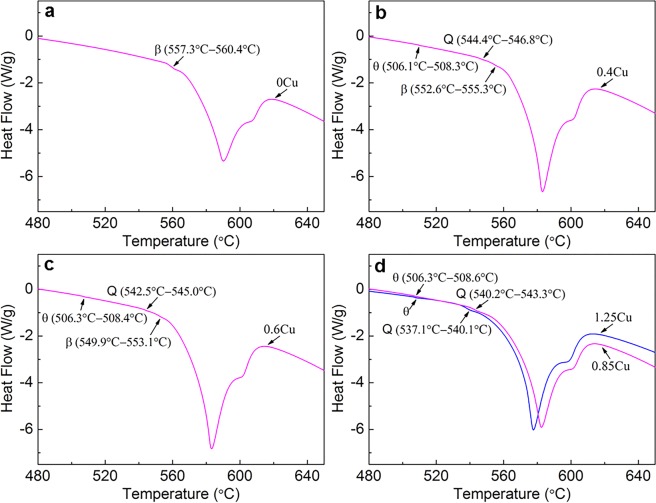
DSC thermal analysis results of the as-cast Al9Si0.5MgCu alloys with different Cu contents, (**a**) 0 wt.% Cu, (**b**) 0.4 wt.% Cu, (**c**) 0.6 wt.% Cu, and (**d**) 0.85 wt.% Cu and 1.25 wt.% Cu.

Figure 5a–d show the DSC thermal analysis curves of the as-cast Al9Si0.5MgCu alloys with 0 wt.% Cu, 0.4 wt.% Cu, 0.6 wt.% Cu, and 0.85 wt.% Cu and 1.25 wt.% Cu, respectively. From Fig. 5a, for the Al9Si0.5MgCu alloy without Cu, the endothermic peak of β intermetallic phase (557.3 °C–560.4 °C) was the only endothermic peak observed for intermetallic phases, indicating that the β phase was the only intermetallic phase in the alloy. After adding Cu, the endothermic peaks of the Cu-containing Q and θ intermetallic phases were observed in the DSC thermal analysis curves, as shown in Fig. 5b–d, indicating the formation of the Q and θ intermetallic phases in the Al9Si0.5MgCu alloys with the presence of Cu. From Fig. 5b,c, the endothermic peak of the β phase was still observed, indicating that the β phase was still existed in the alloys with 0.4 wt.% Cu and 0.6 wt.% Cu. From Fig. 5d, the endothermic peak of the β phase was not observed in the DSC thermal analysis curves of the alloys with 0.85 wt.% Cu and 1.25 wt.% Cu, indicating the disappearance of the β phase in the Al9Si0.5MgCu alloys when the Cu content was increased to 0.85 wt.%. With the increase of Cu content, the temperature range of the endothermic peaks of the β and Q phases decreased gradually, while the temperature range of the endothermic peak of the θ phase was always kept between 506.1 °C and 508.6 °C, indicating the eutectic feature of the θ phase, which agreed well with the general understanding. The DSC thermal analysis results confirmed the disappearance of the β phase in the Al9Si0.5MgCu alloys when the Cu content was increased to 0.85 wt.%, which agreed well with the SEM observation in Fig. 3.

#### Microstructure after heat treatment

**Figure 6:**
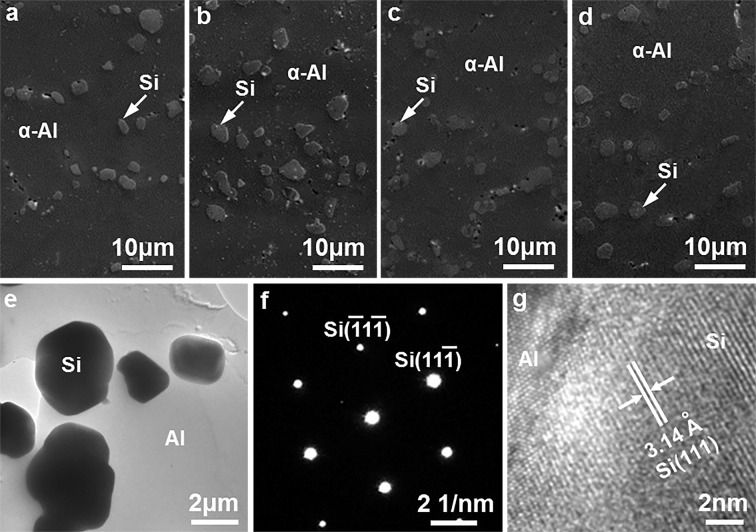
(**a**–**d**) SEM and (**e**–**g**) TEM micrographs showing the microstructure of the T6 heat-treated Al9Si0.5MgCu alloys with different Cu contents, (**a**) SEM of 0 wt.% Cu alloy, (**b**) SEM of 0.4 wt.% Cu alloy, (**c**) SEM of 0.85 wt.% Cu alloy, (**d**) SEM of 1.25 wt.% Cu alloy, **(e**) bright-field TEM image, (**f**) selected area electron diffraction pattern of Si phase, (**g**) high-resolution TEM image.

Figure 6a–d show the microstructure of the cast Al9Si0.5MgCu alloys with different Cu contents under SEM after T6 heat treatment. The primary α–Al phase and the spheroidal Si particles were the two phases observed in the T6 heat-treated alloys under SEM. The spheroidal Si phase in the T6 heat-treated alloys was much similar with each other, with a size of 2–5 µm, and the spheroidal Si phase was determined precisely by TEM in Fig. 6e–g. It was well reported that the fibrous eutectic Si phase in the as-cast Al–Si–Mg–Cu alloys was spheroidized into the spheroidal Si particles after solution treatment^4,7,23^, and the spheroidal Si particles observed in the T6 heat-treated microstructure indicated the well spheroidization of the Si phase after solution treatment. The intermetallic phases were hardly observed in the microstructure of the T6 heat-treated alloys, confirming that the intermetallic phases in the grain boundary of the as-cast alloys were well dissolved into the primary α–Al matrix after solution treatment. Figure 6e presents the bright-field TEM image of the spheroidized Si phase in the T6 heat-treated Al9Si0.5MgCu alloys. The lattice structure of the Si phase is diamond cubic with the unit cell parameter of *a* = 0.5431 nm, and the consequent interplanar spacing of the (111) planes of the Si phase is 0.31355 nm. The selected area electron diffraction pattern shown in Fig. 6f and the high-resolution TEM image shown in Fig. 6g indicated a 0.3140 nm interplanar spacing of the (111) planes of the spheroidal phase shown in Fig. 6e, which agreed well with the interplanar spacing of the (111) planes of the Si phase, and it confirmed that the spheroidal particles observed in the T6 heat-treated alloys were the spheroidized Si phase.

**Figure 7:**
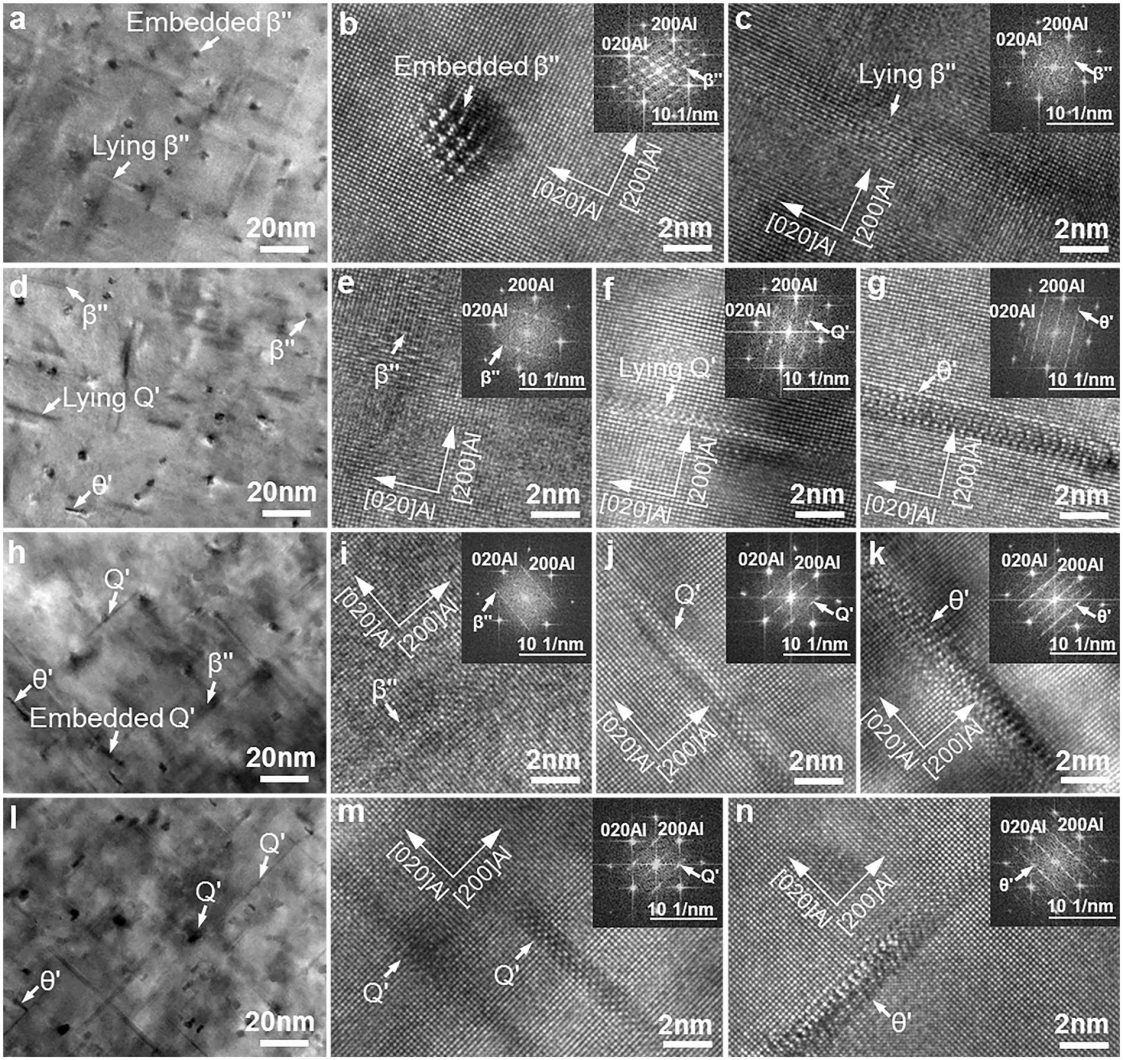
TEM images taken along the <001> Al axis showing the precipitates in the α–Al matrix of the T6 heat-treated Al9Si0.5MgCu alloys with different Cu contents, (**a**–**c**) 0 wt.% Cu, (**d**–**g**) 0.4 wt.% Cu, (**h**–**k**) 0.85 wt.% Cu and (**l**–**n**) 1.25 wt.% Cu. (**a**,**d**,**h**,**l)** bright-field TEM images, (**b**,**c**,**e**–**g**,**i**–**k**,**m**,**n**) high-resolution TEM images.

**Figure 8:**
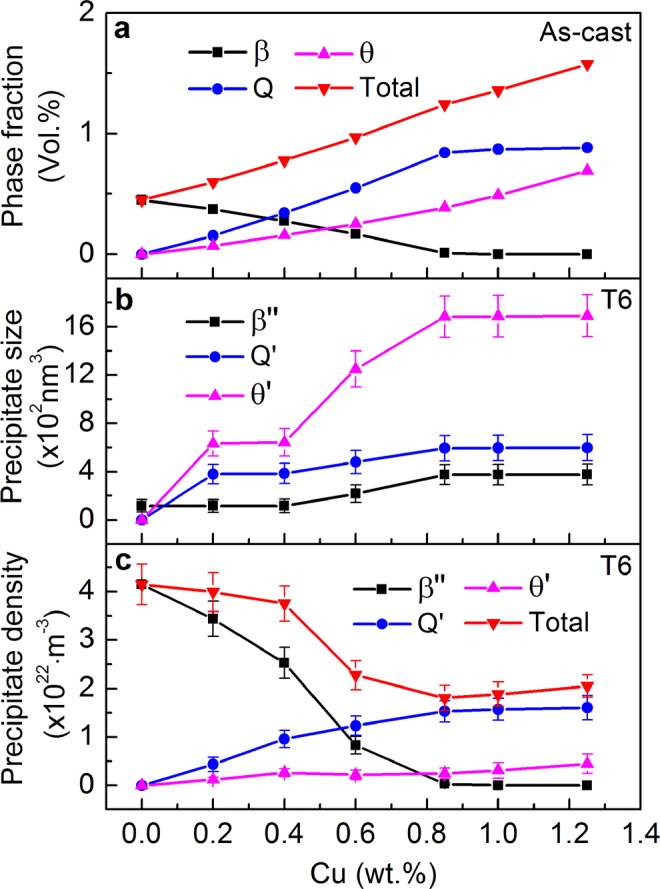
Quantitative results of the (**a**) volume fraction of intermetallic phases in the as-cast Al9Si0.5MgCu alloys and the (**b**) size and (**c**) number density of precipitates in the T6 heat-treated Al9Si0.5MgCu alloys.

After solution treatment, the intermetallic phases in the grain boundary of the as-cast Al9Si0.5MgCu alloys were dissolved into the α–Al matrix and existed in the form of supersaturated solid solution atoms. After ageing treatment, the supersaturated solid solution atoms were precipitated out in the form of nanoscale precipitates. However, it was hard to recognize these nanoscale precipitates in the α–Al matrix under SEM, and TEM was used to identify these nanoscale precipitates. Figure 7 shows the TEM micrographs taken along the <001> Al axis showing the nanoscale precipitates in the α–Al matrix of the Al9Si0.5MgCu alloys after T6 heat treatment. In the alloy without Cu, β″–Mg_2_Si was the only precipitate in the α–Al matrix, as shown by the bright-field TEM image in Fig. 7a. The β″ precipitate was in needle-like shape^2–5^, so embedded β″ precipitate and lying β″ precipitate were observed in the bright-field TEM image, and the embedded and lying β″ precipitates were the same β″ precipitate in nature with different observation directions. Figure 7b,c show the high-resolution TEM images of the embedded and lying precipitates shown in Fig. 7a, and the inserts in Fig. 7b,c show the corresponding fast Fourier transform (FFT) patterns of the precipitates, which verified that the precipitate in the alloy without Cu was β″^2–4,24^. In the alloy with 0.4 wt.% Cu, β″, Q′–Al_5_Cu_2_Mg_8_Si_6_ and θ′–Al_2_Cu precipitates were found in the α–Al matrix, as shown by the bright-field TEM image in Fig. 7d. The Q′ precipitate was in lath shape and the θ′ precipitate was in platelet shape^5,15,25–27^, so embedded and lying Q′ precipitates could be observed while only lying θ′ precipitate could be observed under TEM. Figure 7e–g show the high-resolution TEM images of the precipitates shown in Fig. 7d, and the inserts in these images show the corresponding FFT patterns of the precipitates, which confirmed that the precipitates in the 0.4 wt.% Cu alloy were β″, Q′ and θ′^15,25–27^. Similarly, β″, Q′ and θ′ precipitates were identified in the α–Al matrix of the alloy with 0.85 wt.% Cu, as verified by the bright-field TEM image in Fig. 7h and the high-resolution TEM images in Fig. 7i–k. In the alloy with 1.25 wt.% Cu, β″ precipitate was hardly observed, and Q′ and θ′ precipitates were found in the α–Al matrix, as indicated by the bright-field TEM image in Fig. 7l. The high-resolution TEM images and FFT patterns in Fig. 7m,n confirmed that the precipitates in the 1.25 wt.% Cu alloy were Q′ and θ′. From Fig. 7a,d,h and l, with the increase of Cu content, the volume fraction of the β″ precipitate decreased, while the volume fraction of the Q′ and θ′ precipitates increased. Moreover, the volume fraction of the Q′ precipitate was higher than that of the θ′ precipitate. The quantitative results of the precipitates are shown in Fig. 8b,c.

## Discussion

Generally, the strength of alloys is decided by grain size strengthening, secondary phase strengthening, solution and precipitate strengthening as well as strain strengthening. For the investigated Al9Si0.5MgCu alloys with different Cu contents, the grain sizes of the primary α–Al matrix phase were found no difference to each other under the polarized optical observation, also there was no difference for the secondary Si phase (Fig. 6a–d). From Figs 3 and 6, the difference was the trade-off of the different secondary intermetallic phases in the as-cast alloys and the consequent trade-off the secondary precipitate strengthening phases in the T6 heat-treated alloys, which determined the difference of the strength of the T6 heat-treated alloys. In order to reveal the mechanism of the platform in the strength of the T6 heat-treated Al9Si0.5MgCu alloys, quantitative analysis of the intermetallic phases in as-cast state and the consequent precipitate strengthening phases in T6 heat-treated state was done.

Figure 8a presents the quantitative volume fraction of the intermetallic phases in the as-cast Al9Si0.5MgCu alloys at different Cu contents, which was calculated by the CALPHAD software Pandat. The volume fraction of the β intermetallic phase decreased with increasing Cu content, and reached nearly zero when the Cu content was increased to 0.85 wt.%. Moreover, the volume fractions of the Q and θ intermetallic phases increased nearly linearly with increasing Cu content, but the increase of the Q phase was insignificant when the Cu content was higher than 0.85 wt.%. There was phase trade-off between the β phase and the Q and θ phases with increasing Cu content. The total volume fraction of the β, Q and θ intermetallic phases increased nearly linearly with the increase of Cu content. Figure 8b,c show the quantitative results of the volume size and number density of the precipitates in the T6 heat-treated Al9Si0.5MgCu alloys at different Cu contents, respectively. The determination of the size and number density of precipitates was based on the measurement and statistics from the TEM images. The detail of the determination of the size and number density of precipitates is provided in the supplementary information. With the increase of Cu content, the size of the β″, Q′ and θ′ precipitates was maintained nearly no change till 0.4 wt.% Cu, then was increased till 0.85 wt.% Cu, after kept nearly constant. The size of the precipitates was in the incremental sequence of β″, Q′ and θ′. Meanwhile, with increasing Cu content, the number density of the β′′ precipitate and the Q′ and θ′ precipitates showed trade-off trends, and the number density of the β″ precipitate decreased while that of the Q′ precipitate increased. The number density of the θ′ precipitate increased till 0.4 wt.% Cu, then maintained nearly no change between 0.4 wt.% Cu and 0.85 wt.% Cu, after increased again. The total number density of the β″, Q′ and θ′ precipitates was slightly decreased up to 0.4 wt.% Cu, then was significantly decreased till 0.85 wt.% Cu, followed by a slight increase with the further increase of Cu content.

The precipitate strengthening of the alloy is achieved through the pinning of the dislocation by the precipitates under loading. According to the Orowan theory^28,29^, for the uniform distribution of one kind of precipitate in the matrix, with the given volume fraction of the precipitate, the increase in precipitate size will decrease the precipitate strengthening effect, due to the decrease of the hindering of the movement of dislocation under decreased number density of the precipitate, and vice versa. However, for an individual precipitate, the precipitate with larger size will provide higher precipitate strengthening. The uniformly distributed precipitate strengthening phases of β″, Q′ and θ′ in the Al matrix determined the final value of the yield strength and hardness of the T6 heat-treated Al9Si0.5MgCu alloys. The β″, Q′ and θ′ precipitates are in needle, lath and platelet shapes, respectively^2–5,24–27^, by the principle of minimum energy during precipitation. The size of the β″, Q′ and θ′ precipitates is in the incremental sequence, which is determined by their shapes, therefore the precipitate strengthening of the individual precipitate of β″, Q′ and θ′ is also in the incremental sequence. With the increase of Cu content up to 0.4 wt.%, the size of these three precipitates maintained no change, while the total number density of the precipitates decreased slightly, and the increased number density of the Q′ and θ′ precipitates could provide higher strength increase than the strength decrease resulted from the decreasing number density of the β″ precipitate, which led to the increase of the yield strength and hardness of the T6 heat-treated alloys in this composition range. With the increase of Cu content between 0.4 wt.% and 0.85 wt.%, the size and strengthening effect of the individual β″ precipitate increased slightly, while the number density of the β″ precipitate decreased significantly, and the overall strengthening effect of the β″ precipitate decreased; the size and strengthening effect of the individual Q′ precipitate increased slightly, while the number density of the Q′ precipitate increased notably, and the overall strengthening effect of the Q′ precipitate increased; the size and strengthening effect of the individual θ′ precipitate increased dramatically, while the number density of the θ′ precipitate kept nearly constant, and the overall strengthening effect of the θ′ precipitate increased. Therefore the trade-off the overall strengthening effect of the β″ precipitate and the Q′ and θ′ precipitates maintained the yield strength and hardness of the T6 heat-treated alloys as platform in this composition range. With the increase of Cu content after 0.85 wt.%, Q′ and θ′ replaced β″ for the precipitate strengthening, and the increase of the number density of the Q′ and θ′ precipitates resulted in the increase of the yield strength and hardness of the T6 heat-treated alloys in this composition range.

**Figure 9:**
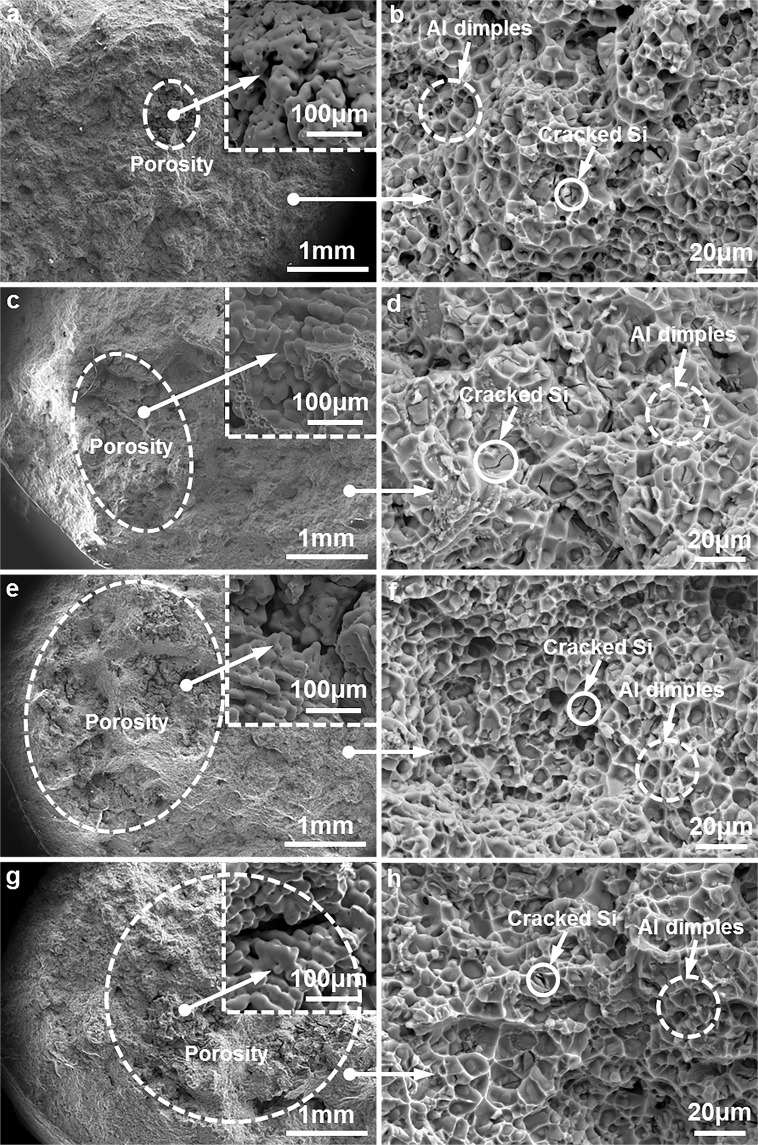
SEM micrographs showing the fracture morphology of the T6 heat-treated Al9Si0.5MgCu alloys with different Cu contents, (**a**,**b**) 0 wt.% Cu, (c,d) 0.4 wt.% Cu, (**e**,**f**) 0.85 wt.% Cu, and (**g**,**h**) 1.25 wt.% Cu.

The above mentioned results and discussion confirmed that the strength of the T6 heat-treated Al9Si0.5MgCu alloys kept as a platform in the composition range between 0.4 wt.% Cu and 0.85 wt.% Cu, and the strength of the T6 heat-treated Al9Si0.5MgCu alloys didn′t enhance monotonously with increasing Cu content in specific composition range. The present industrial practice pursuing the strengthening of the cast Al–Si–Mg–Cu alloys by adding more Cu should be reevaluated. Figure 9 shows the fracture morphology of the T6 heat-treated Al9Si0.5MgCu alloys with different Cu contents under SEM. The defect of porosity can be observed in the fracture of the alloys, as indicated by the dashed circles in Fig. 9a,c,e and g, and these observed porosities are very possibly interconnected in the three dimensional space. The inserts in Fig. 9a,c,e and g show the morphology of the locality of the porosity under higher magnification. From Fig. 9a,c,e and g, the size of porosity in the alloys increased with increasing Cu content. Figure 9b,d,f and h show the high magnification fracture morphology in the non-porosity area of the fracture, which comprised the Al dimples and cracked Si, and there was no difference for the fracture morphology in the non-porosity area of the alloys with different Cu contents. The ductility of the cast alloys was reported depending on the area fraction of the defect in the fracture rather than the bulk porosity content in the alloys^30^, and the increase of porosity area fraction in the fracture contributed to the monotonous decrease of the ductility of the T6 heat-treated alloys with increasing Cu content, as shown in Figs 2b and 9. The low melting point θ phase was formed in the alloys between 506.1 °C and 508.6 °C when Cu was added, as indicated by the DSC results in Fig. 5, which significantly enlarged the solidification temperature range of the alloys, and this led to the difficulty of shrinkage compensation and the increasing tendency of porosity at final stage of solidification. In addition, the size and volume fraction of the θ phase increased with increasing Cu content, as shown in Figs 3 and 8a. Thus the porosity in the alloys increased with increasing Cu content. The increase of precipitation and strength with increasing Cu content also contributed to the decrease of ductility, due to the strength-ductility trade-off dilemma. However, from Fig. 2, with the increase of Cu content from 0 wt.% to 1.25 wt.%, the strength was increased by 7.4%, but the ductility was decreased significantly by 62.5%, so the decrease of ductility of the alloys with increasing Cu content was mainly attributed to the increase of the area fraction of porosity in the fracture. The further increase of Cu content in the platform range of the T6 heat-treated cast Al9Si0.5MgCu alloys is not favorited industrially, as it only decreases ductility without any benefit to the strength enhancement.

## Conclusion

In summary, the phase trade-off induced platform in the strength of the T6 heat-treated cast Al–Si–Mg–Cu alloys with different Cu contents was found and investigated. The hardness and yield strength of the T6 heat-treated Al9Si0.5MgCu alloys kept as a platform in the composition range from 0.4 wt.% Cu to 0.85 wt.% Cu, and increased with increasing Cu content before and after the platform. With increasing Cu content, the β″ precipitation strengthening phase decreased, while the Q′ and θ′ precipitation strengthening phases increased, and the trade-off of these strengthening phases induced the platform in the strength. The ductility of the T6 heat-treated Al9Si0.5MgCu alloys decreased with increasing Cu content due to the increase of the defect of porosity. The strength of the T6 heat-treated cast Al–Si–Mg–Cu alloys is not necessarily increased with increasing Cu content in specific composition range, and the pursuing of the further strengthening of the alloys in this trapped range by adding more Cu content is not favorited industrially, as it only decreases ductility without any benefit to the strength enhancement of the alloys.

## Methods

In experiments, different levels of Cu were added into the Al–9Si–0.5Mg (in wt.%) cast alloy, which was melted at 750 °C in an electric resistance furnace. The melt was degassed through injecting pure argon into the melt by using a rotary degassing impeller. After 0.2 wt.% Al5Ti1B master alloy was added into the melt for grain refinement. The melt was poured at 720 °C into an ASTM B–108 permanent mould preheated at 460 °C, and two ϕ10 mm round tensile test bars with a gauge length of 50 mm were made from each casting. The actual composition of the Al9Si0.5MgxCu alloys were Si 8.8 wt.%, Mg 0.46 wt.%, Fe 0.11 wt.%, Mn 0.06 wt%, Ti 0.14 wt%, Sr 0.014 wt.%, B 0.002 wt.% with Cu at 0.02, 0.21, 0.42, 0.60, 0.85, 1.02, 1.25 wt.% and balanced Al. The cast tensile test bars were subjected to T6 heat treatment, including solution treatment at 540 °C for 8 h for the alloy without Cu, 504 °C for 2 h plus 540 °C for 6 h for the alloys with 0.21 and 0.42 wt.% Cu, and 504 °C for 2 h plus 530 °C for 6 h for the alloys with 0.60, 0.85, 1.02 and 1.25 wt.% Cu, followed by the peak ageing at 190 °C to obtain the maximum strength.

The microstructure was examined using the Zeiss SUPRA 35VP scanning electron microscope (SEM) and the JEOL–2100 F transmission electron microscopy (TEM). The samples for SEM analysis were first polished by the standard method of grinding, and then etched by the 15 vol.% hydrochloric acid for 2 minutes before SEM observation. The specimens for TEM observation were first ground to the thickness of ~100 μm, and then electro-polished using a chemical solution at 20 V and −30 °C. The chemical solution for electropolishing was the nitric acid and methyl alcohol with a ratio of 1:3.TEM was operated at 200 kV for bright-field imaging, high-resolution TEM (HRTEM) imaging and selected area electron diffraction. Vickers hardness tests were conducted at room temperature on a FM–800 tester with an applied load of 10 kg for 10s. Tensile tests were conducted at room temperature using an Instron 5500 tester at a ramp rate of 1 mm/min. Differential scanning calorimetry (DSC) thermal analysis was conducted on the TA instrument Q800 with a heating rate of 10 °C/min.

## Supplementary information

Supplementary Information
