# Supplementary material for: Formation of strength platform in cast Al–Si–Mg–Cu alloys
Source: Sci Rep. 2019 Jul 3;9:9582. doi: 10.1038/s41598-019-46134-7 (PMC6610148; doi:10.1038/s41598-019-46134-7)
Supplement: Supplementary file 1 — Supplementary Information [file 41598_2019_46134_MOESM1_ESM.docx]

**Formation of strength platform in cast Al–Si–Mg–Cu alloys**

# Xixi Dong1, Sajjad Amirkhanlou2, and Shouxun Ji1,*

1Brunel Centre for Advanced Solidification Technology (BCAST), Institute of Materials and Manufacturing, Brunel University London, Uxbridge, UB8 3PH, United Kingdom

2Department of Materials, University of Oxford, Oxford, OX1 3PH, United Kingdom

*shouxun.ji@brunel.ac.uk

**Supplementary Information**

The detail for the determination of the volume size (Fig. 8b) and number density (Fig. 8c) of the β'', Q′ and θ′ precipitates in the cast Al9Si0.5MgCu alloys after solution and peak ageing treatment is as follows:

The β'' and Q′ precipitates are in needle and lath shape respectively^2–5,24–27^, and the volume size of the two precipitates can be obtained by the multiplication of their cross-sectional area and height. From the HRTEM image shown in Fig. 7b, the cross section of the β'' precipitate is in parallelogram shape, and the cross-sectional area of the β'' precipitate was calculated after measuring the side lengths and angle of the parallelogram. The Q′ precipitate was observed in rectangular cross-sectional shape, and its cross-sectional area was calculated in similar way from HRTEM images. From the bright field TEM images, the length of multiple β'' and Q′ precipitates were measured and the statistical average length was used for the calculation of the volume size. The θ′ precipitate is in platelet shape^24–27^, and its volume size was calculated by the multiplication of the cross-sectional area and height of the plate. From the bright field TEM images, the diameters of the circular cross section of multiple θ′ precipitates were measured, and the cross-sectional area of the θ′ precipitate was calculated by the statistical average of the measured diameters, according to the formula of the area of a circle. The height of the platelet θ′ precipitate was measured from the HRTEM images shown in Fig. 7g, k and n. The bright-field TEM view field of 320nm x 210nm was used for the statistical measurement of the length of the β'' and Q′ precipitates and the diameter of the θ′ precipitates, and more than 60 corresponding precipitates were measured to give each of the statistical average measurement values of the β'', Q′ and θ′ precipitates. An example was provided demonstrating the bright-field TEM view field of 320nm x 210nm used for the statistical measurement of the length of the β'' precipitate in the α–Al matrix of the T6 heat-treated Al9Si0.5MgCu alloys with 0 wt.% Cu, as shown in Figure 1S.


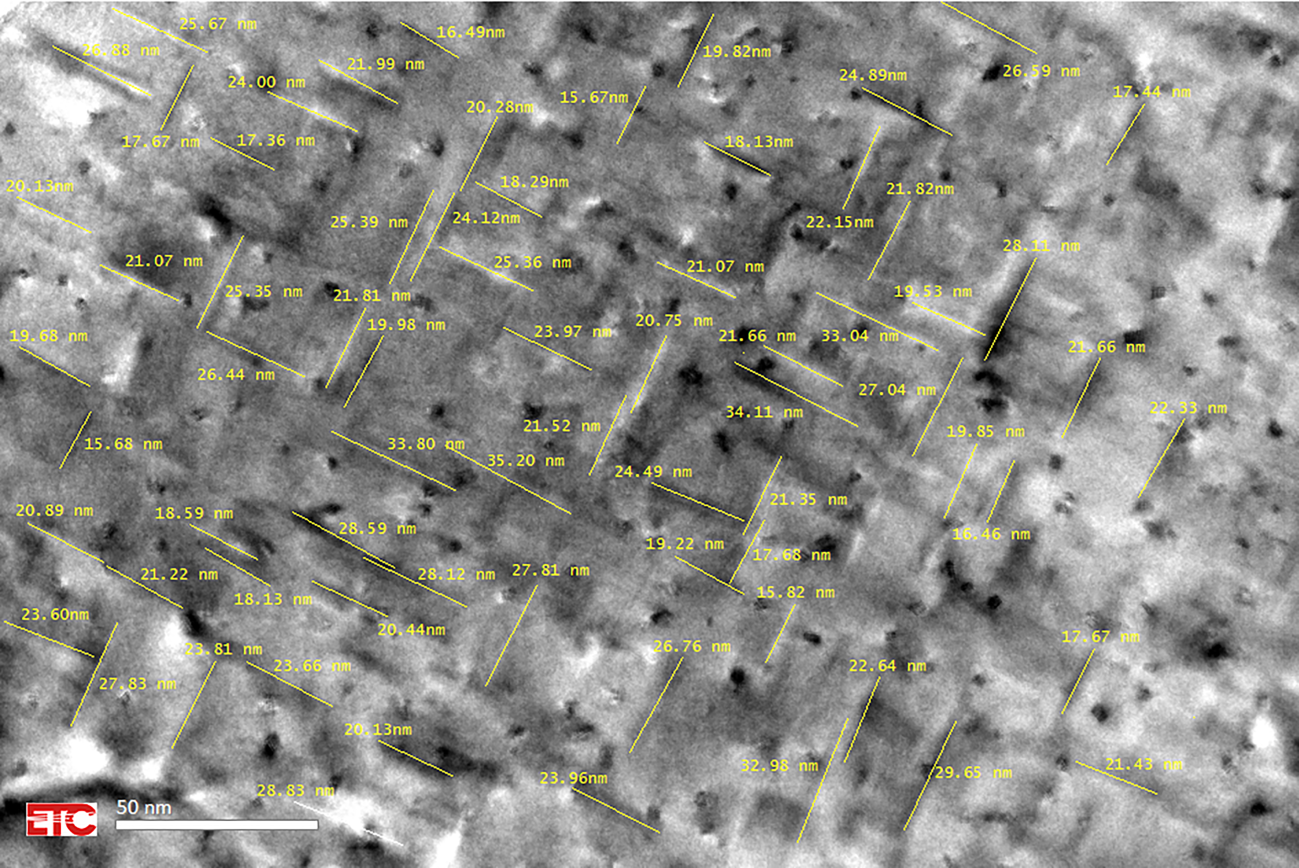


**Figure 1S**. Example showing the bright-field TEM view field of 320nm x 210nm used for the statistical measurement of the length of the β'' precipitate in the α–Al matrix of the T6 heat-treated Al9Si0.5MgCu alloys with 0 wt.% Cu.

The volume fractions of the α–Al phase and the β, Q and θ intermetallic phases in the as-cast Al9Si0.5MgCu alloys were calculated by the CALPHAD software Pandat, as shown in Fig. 8a. From Fig. 6, the β, Q and θ intermetallic phases were completely dissolved into the α–Al phase after solution. From Fig. 7, the dissolved intermetallic phases were precipitated in the α–Al phase in the form of β'', Q′ and θ′ precipitates after ageing. So the volume fraction of the α–Al phase in the solution and aged alloys were supposed to be the sum of the volume fractions of the α–Al phase and the intermetallic phases in the as-cast alloys. The volume fractions of the β'', Q′ and θ′ precipitates in the solution and aged alloys were considered to be the same with that of the β, Q and θ intermetallic phases in the as-cast alloys, respectively. Thus the numbers of the β'', Q′ and θ′ precipitates in the solution and aged alloys were calculated by the division of the volume fraction and the volume size of the precipitates, and the number density of the β'', Q′ and θ′ precipitates in the α–Al phase was calculated by the division of the numbers of the precipitates and the volume fraction of the α–Al phase in the solution and aged alloys.
